# The Relation of Suicide to Alcohol Consumption

**Published:** 1893-08

**Authors:** 


### The Relation of Suicide to Alcohol Consumption.

The *Medical Press and Circular*, March 29, has compiled some striking data concerning the progress of suicide in France. That journal makes a statement from statistics that have been compiled in France; it would seem that suicides in that country bear an important relation to the amount of alcohol consumed. From 1836 to 1840 the consumption of alcohol was upwards of 500,000 hectolitres, and during that period 137 persons committed suicide as the result of alcoholism, and from the same cause there were 226 accidental deaths. From 1880 to 1885, when the consumption of alcohol rose to 1,800,000 hectolitres, there were 868 suicides and 537 accidental deaths, for which alcoholism was responsible—a very notable increase.

The wide gap in the dates chosen for comparison has been necessary for more than one reason, but one important reason was that it seemed best to omit the period of the Franco-Prussian conflict, as being non-comparable by reason of the depressing influences of war and communistic struggles.

TRIONAL, under quite recent clinical tests, is found to have a special value in uncomplicated agrypnia, or wakefulness with a certain amount of excitement. In these cases it is said to have acted promptly and effectively in doses of one to two grammes. Trional is useful also in convalescence from the abuse of cocaine and morphine. Some of the reports state that doses of two grammes would usually procure for these patients a sleep of from seven to nine hours' duration. According to a recent report by Boettiger, dementia with hallucinations was very favorably influenced by trional. The same writer reports thirty-three cases of insomnia with physical disturbances in the insane. These were primary or secondary, or were accompanied by moderate delirium or motor restlessness. Doses of one to two grammes of trional promptly induced a sleep of six to ten hours. The full report of the cases referred to was published in the *Berl. Klin. Woch.*, No. 42, 1892.

Recent Observations on Sulfonal.—In a recent number of the Birmingham *Medical Review*, Dr. T. Sidney Short states that in his clinical experiences with sulfonal he often obtained a more rapid action from the drug than he had supposed it to possess. Doses of only fifteen grains sometimes produced sleep in a very few minutes, and he cites five cases in which the same quanity caused sleep in fifteen minutes. He writes: "I cannot point to any derangement of respiration, circulation, or appetite as following its use, nor any case of cyanosis." Dr. Harding (*Medical World*, March, 1892) writes: "I have used sulfonal in an extensive country practice, and find that it produces natural sleep and has no other action; in sulfonal we have a safe hypnotic that can be administered to the aged as well as to those suffering from organic diseases, and it works admirably with children. Sulfonal will quiet the irritability of teething; it will often prevent convulsions and relieve nervous excitement, and it induces a peaceful sleep. Sulfonal does not constipate, depress the system, or impair the appetite; it leaves no sequelæ, and is accompanied by none of those phenomena sure to accompany the administration of opium, chloral, and the bromides."

